# Enhancement of Astroglial Aerobic Glycolysis by Extracellular Lactate-Mediated Increase in cAMP

**DOI:** 10.3389/fnmol.2018.00148

**Published:** 2018-05-08

**Authors:** Nina Vardjan, Helena H. Chowdhury, Anemari Horvat, Jelena Velebit, Maja Malnar, Marko Muhič, Marko Kreft, Špela G. Krivec, Saša T. Bobnar, Katarina Miš, Sergej Pirkmajer, Stefan Offermanns, Gjermund Henriksen, Jon Storm-Mathisen, Linda H. Bergersen, Robert Zorec

**Affiliations:** ^1^Laboratory of Neuroendocrinology - Molecular Cell Physiology, Institute of Pathophysiology, Faculty of Medicine, University of Ljubljana, Ljubljana, Slovenia; ^2^Laboratory of Cell Engineering, Celica Biomedical, Ljubljana, Slovenia; ^3^Department of Biology, Biotechnical Faculty, University of Ljubljana, Ljubljana, Slovenia; ^4^Laboratory for Molecular Neurobiology, Institute of Pathophysiology, Faculty of Medicine, University of Ljubljana, Ljubljana, Slovenia; ^5^Department of Pharmacology, Max Planck Institute for Heart and Lung Research, Bad Nauheim, Germany; ^6^Nuclear and Energy Physics, Department of Physics, The Faculty of Mathematics and Natural Sciences, University of Oslo, Oslo, Norway; ^7^Norwegian Medical Cyclotron Centre Ltd., Oslo, Norway; ^8^Division of Anatomy, Department of Molecular Medicine, CMBN/SERTA Healthy Brain Ageing Centre, Institute of Basic Medical Sciences, Faculty of Medicine, University of Oslo, Oslo, Norway; ^9^Institute of Oral Biology, Faculty of Dentistry, University of Oslo, Oslo, Norway; ^10^Center for Healthy Aging, Faculty of Health and Medical Sciences, University of Copenhagen, Copenhagen, Denmark

**Keywords:** astrocytes, aerobic glycolysis, L-lactate receptor, cAMP, L-lactate

## Abstract

Besides being a neuronal fuel, L-lactate is also a signal in the brain. Whether extracellular L-lactate affects brain metabolism, in particular astrocytes, abundant neuroglial cells, which produce L-lactate in aerobic glycolysis, is unclear. Recent studies suggested that astrocytes express low levels of the L-lactate GPR81 receptor (EC_50_ ≈ 5 mM) that is in fat cells part of an autocrine loop, in which the G_i_-protein mediates reduction of cytosolic cyclic adenosine monophosphate (cAMP). To study whether a similar signaling loop is present in astrocytes, affecting aerobic glycolysis, we measured the cytosolic levels of cAMP, D-glucose and L-lactate in single astrocytes using fluorescence resonance energy transfer (FRET)-based nanosensors. In contrast to the situation in fat cells, stimulation by extracellular L-lactate and the selective GPR81 agonists, 3-chloro-5-hydroxybenzoic acid (3Cl-5OH-BA) or 4-methyl-*N*-(5-(2-(4-methylpiperazin-1-yl)-2-oxoethyl)-4-(2-thienyl)-1,3-thiazol-2-yl)cyclohexanecarboxamide (Compound 2), like adrenergic stimulation, elevated intracellular cAMP and L-lactate in astrocytes, which was reduced by the inhibition of adenylate cyclase. Surprisingly, 3Cl-5OH-BA and Compound 2 increased cytosolic cAMP also in GPR81-knock out astrocytes, indicating that the effect is GPR81-independent and mediated by a novel, yet unidentified, excitatory L-lactate receptor-like mechanism in astrocytes that enhances aerobic glycolysis and L-lactate production via a positive feedback mechanism.

## Introduction

Aerobic glycolysis, non-oxidative metabolism of glucose despite the presence of adequate levels of oxygen, a phenomenon termed “the Warburg effect,” is an inefficient way to generate energy in the form of ATP. The advantage of this process, however, appears to be in providing intermediates for the biosynthesis of lipids, nucleic acids, and amino acids (Vander Heiden et al., 2009). These are needed for making new cells during division, such as in cancer cells and in developing normal cells, and for cells engaged in morphological reshaping, such as neural cells in the central nervous system (CNS; Goyal et al., 2014).

At least in the brain, aerobic glycolysis appears to be regulated. For example, during alerting, sensory stimulation, exercise, and pathophysiological conditions, L-lactate production and release are upregulated. Although it is still unclear how this takes place at the cellular level, the process likely involves noradrenergic neurons from *locus coeruleus* (LC; Dienel and Cruz, 2016). L-lactate can be produced from glycogen stored in astrocytes, an abundant glial cell type in the CNS, and can be used by neurons and oligodendrocytes, which seem to require L-lactate in addition to D-glucose for their optimal function, including memory formation (Cowan et al., 2010; Barros, 2013; Gao et al., 2016; Dong et al., 2017), myelin production, and the sustenance of long axons (Lee et al., 2012; Rinholm and Bergersen, 2012). In addition, L-lactate is considered to be neuroprotective against various types of brain damage (Castillo et al., 2015) and is required for cancer cell survival (Roland et al., 2014).

These effects suggest that L-lactate not only acts as a fuel but also has extracellular signaling roles (Chen et al., 2005; Barros, 2013; Roland et al., 2014). L-lactate modulates the activity of primary cortical neurons through a receptor-mediated pathway (Bozzo et al., 2013), and the activation of astrocytes by LC neurons results in the release of L-lactate, which back-excites LC neurons and stimulates the further release of noradrenaline (NA; Tang et al., 2014). These effects are supported by the observation that the monocarboxylate transporter 2 (MCT2), transporting L-lactate, is selectively co-located with glutamate receptors at the postsynaptic membranes of fast-acting excitatory synapses (Bergersen et al., 2001). Interestingly, L-lactate appears to promote gene expression that mediates *N*-methyl-D-aspartate (NMDA)-related neuronal plasticity (Yang et al., 2014) and the expression of membrane metabolite receptors at the plasma membrane (Castillo et al., 2015). L-lactate is known to mediate cerebral vasodilatation, causing increased brain blood flow (Gordon et al., 2007, 2008).

The notion of multiple signaling roles of L-lactate and its widespread diffusion in tissues led to the concept of L-lactate being a “volume transmitter” of metabolic information (Chen et al., 2005) and perhaps also a gliotransmitter (Tang et al., 2014) in the brain.

L-lactate signaling may occur through several mechanisms, including the modulation of prostaglandin action (Gordon et al., 2008), redox regulation (Brooks, 2009), and the activation of the L-lactate-sensitive receptors, such as G_i_-protein coupled receptors GPR81 (Lauritzen et al., 2014) or the yet unidentified plasma membrane receptors (Tang et al., 2014).

The L-lactate selectivity of the GPR81 receptor (EC_50_ ≈ 5 mM for rat GPR81; Liu et al., 2009), also known as hydroxycarboxylic acid receptor 1 (HCA_1_ or HCAR1), was discovered in adipose tissues (reported EC_50_ range from 1 to 5 mM; Cai et al., 2008; Liu et al., 2009), where GPR81 is highly expressed and down-regulates the formation of cytosolic cyclic adenosine monophosphate ([cAMP]_i_) by coupling to G_i_-protein and inhibiting the cAMP producing enzyme adenylate cyclase (AC), thereby in an autocrine loop inhibiting lipolysis and promoting energy storage (Ahmed et al., 2010). Whether a similar signaling loop is present in astrocytes is not known and was explored in this study. Quantitative RT-PCR analysis of human, rat, and mouse brain tissue revealed the presence of mRNA GPR81 in the brain, although at very low levels compared to adipose tissue (Liu et al., 2009). Consistent with RT-PCR analysis, RNA sequencing transcriptome databases revealed the presence of GPR81 mRNA in individual types of mouse brain cells, including astrocytes (Zhang et al., 2014; Sharma et al., 2015). Immunohistochemical studies on mouse brain tissue slices also suggested the presence of the GPR81 receptor in neurons, endothelial cells, and at low density of expression in astrocytes, in particular in membranes of perivascular astrocytic processes and not so much in perisynaptic processes of astrocytes (Lauritzen et al., 2014; Morland et al., 2017). The mechanism of how L-lactate via activation of a GPR81 receptor would modulate [cAMP]_i_, cytosolic levels of D-glucose ([glucose]_i_) and L-lactate ([lactate]_i_) in brain, in particular in astrocytes that are actively involved in the regulation of brain metabolism and produce L-lactate, is currently unknown.

In contrast to the situation in adipocytes, where the GPR81 receptor agonist decreases [cAMP]_i_ (Ahmed et al., 2010), we show here by fluorescence resonance energy transfer (FRET) microscopy on single astrocytes expressing FRET-nanosensors for cAMP and L-lactate that very high levels of extracellular L-lactate and the agonist for the L-lactate GPR81 receptor 3-chloro-5-hydroxybenzoic acid (3Cl-5OH-BA; Dvorak et al., 2012) elevate [cAMP]_i_ and [lactate]_i_ in astrocytes, as does the activation of adrenergic receptors (ARs). The 3Cl-5OH-BA-dependent elevation in [lactate]_i_ and the extracellular L-lactate-mediated rise in [cAMP]_i_, both act through the activation of AC in astrocytes, as demonstrated by the use of an AC inhibitor. Interestingly, in astrocytes from the L-lactate specific GPR81 receptor knock-out (KO) mice, 3Cl-5OH-BA still elevated [cAMP]_i_, indicating that the supposedly selective GPR81 agonist also activates a second, yet unidentified excitatory L-lactate receptor-like mechanism. Pretreatment of rat astrocytes with the sub-effective doses of 3Cl-5OH-BA reduced the L-lactate-induced elevation in [cAMP]_i_, suggesting that 3Cl-5OH-BA and L-lactate at least to some extent bind to the same yet unidentified receptor. A new generation GPR81 selective high affinity agonist lead compound, Compound 2 (4-methyl-N-(5-(2-(4-methylpiperazin-1-yl)-2-oxoethyl)-4-(2-thienyl)-1,3-thiazol-2-yl)cyclohexanecarboxamide; Sakurai et al., 2014) reproduced the cAMP enhancing effects of L-lactate and 3Cl-5OH-BA, further supporting the existence of an unidentified L-lactate receptor. The new excitatory L-lactate receptor-mediated mechanism (“metabolic excitability”) may participate in maintaining high [lactate]_i_ in cells exhibiting aerobic glycolysis, such as in astrocytes (Mächler et al., 2016), contributing to the elevated levels of extracellular L-lactate in comparison to the plasma levels (Abi-Saab et al., 2002). While generating metabolic intermediates required for cell division and morphological plasticity, this regulation presumably facilitates the exit of L-lactate into the extracellular space, where it can become an autocrine and paracrine signal. Since relatively high concentrations of L-lactate (20 mM, but not 2 mM) are required for the increase in second messenger cAMP (the predicted brain physiological concentrations of L-lactate are up to 2 mM), the putative novel facilitatory L-lactate receptor-like mechanism may have a role under conditions of very high extracellular L-lactate that may occur during extreme exercise (Osnes and Hermansen, 1972; Matsui et al., 2017) or neuronal activity (for example during seizures; During et al., 1994). Such a scenario may also be relevant if local fluctuations of extracellular L-lactate concentration exist in brain microdomains (Morland et al., 2015; Mosienko et al., 2015).

## Materials and Methods

### Cell Culture, Transfection, and Reagents

Primary cultures of astrocytes were prepared from cortices of 2–3 and 1–4 days old rat and wild type (WT) or GPR81 KO mice pups, respectively, as described previously (Schwartz and Wilson, 1992), and grown in high-glucose (25 mM) Dulbecco’s Modified Eagle’s Medium containing 10% fetal bovine serum, 1 mM sodium pyruvate, 2 mM L-glutamine, and 25 μg/ml penicillin-streptomycin at 37°C in 95% air–5% CO_2_ until reaching 70–80% confluency. Confluent cultures were shaken at 225 rpm overnight, and the medium was changed the next morning; this was repeated three times. After the third overnight shaking, the cells were trypsinized and put in flat tissue culture tubes with 10 cm^2^ growth area. After reaching confluency, the cells were trypsinized and plated on 22-mm diameter glass cover slips coated with poly- L-lysine. By using quantitative PCR, we verified that astrocytes from the GPR81 KO animals were devoid of the GPR81 RNA transcript. The purity of rat astrocytes was determined immunocytochemically using antibodies against the astrocytic marker GFAP (Abcam, Cambridge, United Kingdom) and >94% of imaged cultured cells were GFAP-positive (Stenovec et al., 2016). 3T3-L1 fibroblasts (ATCC-LGC Standards, VA, United States) were grown in high-glucose Dulbecco’s Modified Eagle’s Medium containing 10% fetal bovine serum and 2 mM L-glutamine. BT474 cancer cells (BT-474 Clone 5; ATCC^®^ CRL-3247^TM^; ATCC-LGC Standards) were grown in Hybri-Care medium (ATCC^®^ 46-X^TM^; ATCC-LGC Standards) supplemented with 1.5 g/L sodium bicarbonate and 10% fetal bovine serum. 3T3-L1 and BT474 cells were grown in culture flasks with 25 cm^2^ growth area at 37°C in 95% air–5% CO_2_. After reaching 70–80% confluency, cells were trypsinized and plated on 22-mm diameter glass cover slips coated with poly- L-lysine.

After 24–72 h, transfection with plasmids carrying FRET-based nanosensors was performed with FuGENE^®^ 6 transfection reagent according to manufacturer’s instructions (Promega Co., Madison, WI, United States). Transfection medium contained no serum or antibiotics.

GPR81 KO and WT mice (Ahmed et al., 2010) in C57Bl/6N background were bred in Oslo from founder mice obtained from Stefan Offermanns, Max-Planck-Institute for Heart and Lung Research, Department of Pharmacology, D-61231 Bad Nauheim, Germany. The experimental animals were cared for in accordance with the International Guiding Principles for Biomedical Research Involving Animals developed by the Council for International Organizations of Medical Sciences and the Animal Protection Act (Official Gazette RS, No. 38/13). The experimental protocol was approved by The Administration of the Republic of Slovenia for Food Safety, Veterinary and Plant Protection (Republic of Slovenia, Ministry of Agriculture, Forestry and Food, Ljubljana), Document No. U34401-47/2014/7, signed by Barbara Tomše, DVM. All experiments were performed on rat astrocytes isolated from at least two different animals. All chemicals were from Sigma-Aldrich (St. Louis, MO, USA) unless otherwise noted.

### FRET Measurements of [cAMP]_i_ and PKA Activity

Cells expressing the FRET-based cAMP nanosensor Epac1-camps (Nikolaev et al., 2004) or the PKA nanosensor AKAR2 (Zhang et al., 2005) were examined 24–48 h after transfection with a Plan NeoFluoar 40×/1.3 NA oil differential interference contrast (DIC) immersion objective (Carl Zeiss, Jena, Germany) using a Zeiss LSM510 META confocal microscope (Carl Zeiss, Jena, Germany). Cells were excited at 458 nm, and images (512 × 512) were acquired every 3.5 s using lambda stack acquisition. Emission spectra were collected from the META detector in 8 channels (lambda stack) ranging from 470 nm to 545 nm, each with a 10.7-nm width. Two-channel [cyan fluorescent protein (CFP) and yellow fluorescent protein (YFP)] images were generated using the analytical software Extract channels (Zeiss LSM510 META, Carl Zeiss, Jena, Germany). Channels with emission spectra at 470 and 481 nm and emission spectra at 513, 524, and 534 nm were extracted to the CFP and YFP channels, respectively. YFP and CFP fluorescence intensities were quantified within a region of interest (ROI) selected for individual cells expressing Epac1-camps or AKAR2 using the LSM510 META software.

In some experiments, astrocytes expressing the Epac1-camps FRET nanosensor, were examined 24 h after transfection with a fluorescence microscope Zeiss Axio Observer.A1 (Zeiss, Oberkochen, Germany) with a CCD camera and monochromator Polychrome V (Till Photonics, Graefelfing, Germany) as a monochromatic source of light with a wavelength 436 nm/10 nm. Dual emission intensity ratios were recorded using an image splitter (Optical Insights, Tucson, AZ, United States) and two emissions filters (465/30 nm for CFP and 535/30 nm for YFP). Images were acquired every 3.5 s with an exposure time of 0.1 s. CFP and YFP fluorescence intensities were obtained from the integration of ROI over the entire cell using Life Acquisition software (Till Photonics, Graefelfing, Germany).

In the graphs, the FRET ratio signal was reported as the ratio of the CFP/YFP (Epac1-camps) and YFP/CFP (AKAR2) fluorescence signals after subtracting the background fluorescence from the signals using Excel (Microsoft, Seattle, WA, United States). The values of the FRET signals were normalized to 1.0. An increase in the FRET ratio signal reflects an increase in the [cAMP]_i_ and the PKA activity.

Initially, astrocytes were kept in extracellular solution (10 mM Hepes/NaOH, pH 7.2, 10 mM D-glucose, 131.8 mM NaCl, 1.8 mM CaCl_2_, 2 mM MgCl_2_, and 5 mM KCl) or extracellular solution with sodium bicarbonate (10 mM Hepes/NaOH, pH 7.2, 10 mM D-glucose, 131.8 mM NaCl, 1.8 mM CaCl_2_, 2 mM MgCl_2_, 5 mM KCl, 0.5 mM NaH_2_PO_4_ × H_2_O, and 5 mM NaHCO_3_) and were then treated with various reagents following a 100-s baseline: 1 μM NA, 10 μM isoprenaline (ISO), 2 or 20 mM L-lactate (osmolality was adjusted by lowering NaCl in extracellular solution containing sodium bicarbonate) and 0.5 mM 3Cl-5OH-BA. In some experiments cell were after initial a 100-s baseline pretreated with 100 μM 2′,5′-dideoxyadenosine (DDA) or 50 μM 3Cl-5OH-BA for 450 s and then stimulated with 20 mM L-lactate in the presence of DDA or 3Cl-5OH-BA, respectively. The 4-methyl-*N*-(5-(2-(4-methylpiperazin-1-yl)-2-oxoethyl)-4-(2-thienyl)-1,3-thiazol-2-yl)cyclohexanecarboxamide (Compound 2; Sakurai et al., 2014) was custom synthesized (ABX advanced biochemical compounds, D-01454 Radeberg, Germany). After the initial 90–100-s baseline, WT and GPR81 KO astrocytes were treated with GPR81 receptor agonist 3Cl-5OH-BA (0.5 mM) or Compound 2 (50 nM) and recorded for another 300 s. In control experiments, astrocytes were treated only with extracellular solution (Vehicle). Extracellular solution osmolality was ∼300 mOsm, measured with a freezing point osmometer (Osmomat030, Gonotech GmbH, Germany).

### FRET Measurements of [glucose]_i_ and [lactate]_i_

Astrocytes, 3T3-L1 embryonic preadipocyte fibroblast cells and BT474 cancer cells expressing the FRET-based glucose nanosensor FLII^12^Pglu-700 μδ6^1^ (Takanaga et al., 2008; Prebil et al., 2011) or the FRET-based lactate nanosensor Laconic (San Martin et al., 2013) were examined 16–48 h after transfection with a fluorescence microscope (Zeiss Axio Observer.AI, Zeiss, Oberkochen, Germany), with a CCD camera and monochromator Polychrome V (Till Photonics, Graefelfing, Germany) as a monochromatic source of light with a wavelength 436 nm/10 nm. Dual emission intensity ratios were recorded using an image splitter (Optical Insights, Tucson, AZ, United States) and two emission filters; 465/30 nm for ECFP or mTFP and 535/30 nm for EYFP or Venus. Images were acquired every 10 s with an exposure time of 0.1 s. The background fluorescence was subtracted from individual EYFP or Venus and ECFP or mTFP fluorescence signals. The FRET ratio signals, EYFP/ECFP (FLII^12^Pglu-700 μδ6) and mTFP/Venus (Laconic), were obtained from the integration of the ratio signal over the entire cell using Life Acquisition software (Till Photonics, Graefelfing, Germany). The values of the FRET signals were normalized to 1.0. An increase in the FRET ratio signal reflects increases in the [glucose]_i_ and [lactate]_i_.

Initially, cells were kept in extracellular solution with sodium bicarbonate (10 mM Hepes/NaOH, pH 7.2, 3 mM D-glucose, 135.3 mM NaCl, 1.8 mM CaCl_2_, 2 mM MgCl_2_, 5 mM KCl, 0.5 mM NaH_2_PO_4_ × H_2_O, and 5 mM NaHCO_3_), and were then treated with various reagents following a 200-s baseline: 200 μM ISO, 200 μM NA, 2 or 20 mM L-lactate, 0.5 mM 3Cl-5OH-BA, and 6 mM α-cyano-4-hydroxycinnamate (CHC). In some experiments, cells were, after an initial 200 s-baseline, pretreated with 100 μM DDA for 450 s and then stimulated with 0.5 mM 3Cl-5OH-BA in the presence of DDA. Extracellular solution osmolality was ∼300 mOsm, measured with a freezing point osmometer (Osmomat030, Gonotech GmbH, Germany).

### Extraction of mRNA and Quantitative Real-Time PCR (qPCR)

Rat, WT mouse, and GPR81 KO mouse astrocytes were cultured in six-well plates. Total RNA was extracted from cultured astrocytes with the RNeasy Mini Plus Kit (Qiagen, Hilden, Germany). cDNA was synthesized from total RNA using the High-Capacity cDNA Reverse Transcription Kit (Applied Biosystems, Thermo Fisher Scientific, Vilnius, Lithuania). qPCR was performed on ABI PRISM SDS 7500 (Applied Biosystems, Thermo Fisher Scientific) in a 96-well format using TaqMan Universal PCR Master Mix (Applied Biosystems, Thermo Fisher Scientific, Foster City, United States) and gene expression assays for GPR81 (Rn03037047_sH) and 18S rRNA (TaqMan Endogenous Control). Standard quality controls were performed in line with the MIQE Guidelines (Bustin et al., 2009). Expression level of GPR81 mRNA was calculated as gene expression ratio (GPR81 mRNA/18S rRNA) according to the equation: $E_{18SrRNA}^{Ct,18S\,\;rRNA}/E_{GPR81}^{Ct,GPR81}$, where *E* is the PCR efficiency and C_t_ is the threshold cycle for the reference (18S rRNA) or the target (GPR81) gene (Ruijter et al., 2009; Tuomi et al., 2010). PCR efficiency was estimated using the LinRegPCR program (Ruijter et al., 2009; Tuomi et al., 2010).

### Statistical Analysis

Single-exponential increase to maximum functions [*F* = *F*_0_ + *c* × (1 - exp(-*t*/τ))] were fitted to the diagrams with FRET ratio signals using SigmaPlot. The time constant (τ) and the FRET ratio signal amplitudes (*c*) were determined from the fitted curves. *F* is the FRET ratio signal at time *t*, *F*_0_ is the baseline FRET ratio signal, *c* is FRET ratio signal amplitude of *F* -*F*_0_, and τ is the time constant of the individual exponential component. In some experiments, the initial rate of the FRET signal increase (ΔFRET/Δtime) after the addition of various reagents was calculated as the slope of the linear regression function [ΔFRET (%) = slope (%/min) × Δtime (min)] fitting the initial FRET signal decrease or increase. In these experiments, the amplitude of the ΔFRET (%) was determined by subtracting the mean FRET ratio signal of the first 100 s from the last 100 s upon stimulation or *vice versa*, if the FRET signal increased.

The average traces of the predominant responses are presented in the figures for individual stimuli; all other responses are listed in **Table 1**. Unless stated otherwise, the Student’s *t*-test was performed to determine statistical significance. *P* < 0.05 was considered to be significant.

**Table 1:** Responsiveness of astrocytes to adrenergic and L-lactate receptor activation.

FRET nanosensor		Stimulus	*n* (%)	*n* (%)	*n* (%)	*n* (%)	*n*
			increase	decrease	transient decrease	unresponsive	all
**Rat astrocytes**							
cAMP		ISO (10 μM)	8 (100%)	0 (0%)	0 (0%)	0 (0%)	8
		NA (1 μM)	16 (100%)	0 (0%)	0 (0%)	0 (0%)	16
		L-lactate (20 mM)	7 (36.8%)	3 (15.8%)	0 (0%)	9 (47.4%)	19
		3Cl-5OH-BA (0.5 mM)	9 (42.9%)	2 (9.5%)	0 (0%)	10 (47.6%)	21
PKA		L-lactate (2 mM)	0 (0%)	0 (0%)	0 (0%)	7 (100%)	7
		L-lactate (20 mM)	8 (57.1%)	1 (7.1%)	0 (0%)	5 (35.7%)	14
		3Cl-5OH-BA (0.5 mM)	9 (56.3%)	2 (12.5%)	0 (0%)	5 (31.3%)	16
Glucose		ISO (200 μM)	5 (16.1%)	0 (%)	0 (0%)	26 (83.9%)	31
		NA (200 μM)	8 (40%)	0 (%)	0 (0%)	12 (60%)	20
		L-lactate (2 mM)	0 (0%)	0 (0%)	0 (0%)	11 (100%)	11
		L-lactate (20 mM)	3 (15%)	4 (20.0%)	13 (65.0%)	0 (0%)	20
		3Cl-5OH-BA (0.5 mM)	1 (5.3%)	13 (68.4%)	1 (5.3%)	4 (21.1%)	19
Lactate		ISO (200 μM)	10 (63%)	0 (0%)	0 (0%)	6 (27%)	16
		NA (200 μM)	8 (88.9%)	0 (0%)	0 (0%)	1 (11.1%)	9
		L-lactate (20 mM)	14 (93.3%)	0 (0%)	0 (0%)	1 (6.7%)	15
		3Cl-5OH-BA (0.5 mM)	14 (100%)	0 (0%)	0 (0%)	0 (0%)	14
**Mouse astrocytes**							
cAMP	WT	3Cl-5OH-BA (0.5 mM)	11 (68.8)	0 (0)	0 (0)	5 (31.2)	16
		Compound 2 (50 nM)	10 (66.7%)	0 (0)	0 (0)	5 (33.3%)	15
	KO GPR81	3Cl-5OH-BA (0.5 mM)	11 (73.3)	0 (0)	0 (0)	4 (26.7)	15
		Compound 2 (50 nM)	11 (100%)	0 (0)	0 (0)	0 (0)	11


*ISO, isoprenaline; NA, noradrenaline; 3Cl-5OH-BA, 3-chloro-5-hydroxybenzoic acid; Compound 2, 4-methyl-*N*-(5-(2-(4-methylpiperazin-1-yl)-2-oxoethyl)-4-(2-thienyl)-1,3-thiazol-2-yl)cyclohexanecarboxamide; *n*, number of cells studied. The increase or decrease in the FRET ratio signal was set by determining a threshold of 3 standard deviations of the average signal measured prior to the application of agents (baseline), as described (Horvat et al., 2016). The frequency of unresponsive cells did not differ in KO group compared to WT group, when treated with 3Cl-5OH-BA (*P* = 0.78). The frequency of unresponsive cells was significantly lower in KO group compared to WT group, when treated with Compound 2 (*P* = 0.03; chi-square statistic).*

## Results

We studied here how extracellular L-lactate and agonists of the L-lactate GPR81 receptor affect cAMP signaling and changes in the intracellular levels of metabolites in isolated astrocytes, which can express the GPR81 receptor (Lauritzen et al., 2014; Sharma et al., 2015) and mRNA for GPR81 can be measured in these cells (Supplementary Figure S1). Real-time FRET imaging of cultured cortical astrocytes, expressing FRET-nanosensors for cAMP, glucose, and lactate was performed.

### Extracellular L-Lactate and GPR81 Lactate Receptor Agonists Increase [cAMP]_i_ in Astrocytes

**FIGURE 1:**
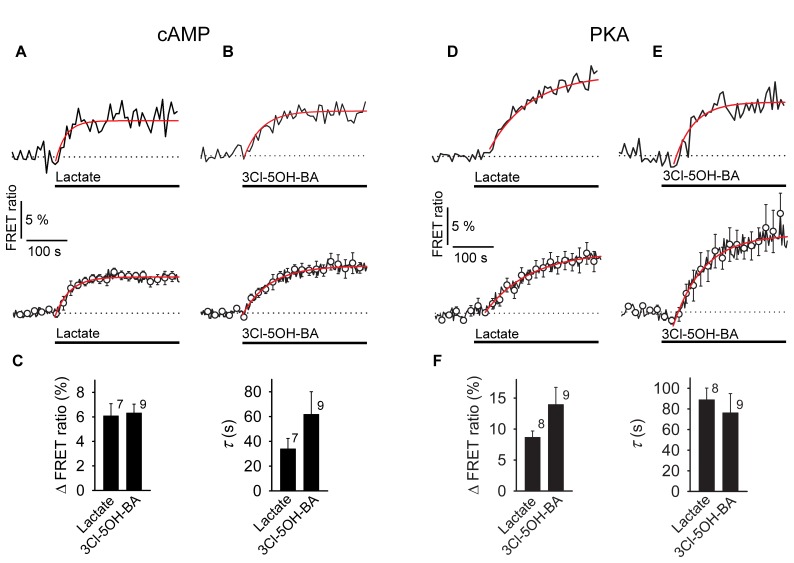
Application of L-lactate and 3Cl-5OH-BA increases [cAMP]_i_ and the intracellular PKA activity in rat astrocytes. **(A,B)** Representative (above) and mean time-course (below) of the Epac1-camps FRET ratio signal upon the addition of **(A)** L-lactate (20 mM) and **(B)** 3Cl-5OH-BA (0.5 mM), a selective agonist of the GPR81 receptor. Data are expressed as percentages of the inverse FRET ratio signal (CFP/YFP) relative to the baseline values. Single exponential rise functions were fitted to the curves: (**A**, above) *FRET ratio* = [0.98 ± 0.01] + [0.06 ± 0.01] × (1 – exp(–*t*/[25.00 ± 5.63 s])), (**A**, below) *FRET ratio* = [1.00 ± 0.00] + [0.05 ± 0.00] × (1 – exp(–*t*/[ 35.71 ± 2.55 s])), and (**B**, above) *FRET ratio* = [0.94 ± 0.00] + [0.06 ± 0.01] × (1 – exp(–*t*/[41.49 ± 5.85 s])), (**B**, below) *FRET ratio* = [1.00 ± 0.00] + [0.07 ± 0.00] × (1 – exp(*t*/[66.67 ± 4.42 s])). Note that the addition of L-lactate and 3Cl-5OH-BA increased the FRET ratios, indicating increases in [cAMP]_i_. Each data point represents the mean ± s.e.m. **(C)** Mean changes in the FRET ratio (ΔFRET ratio) and mean time-constants (τ) upon L-lactate and 3Cl-5OH-BA stimulation. Changes in FRET ratio are expressed as percentages relative to the initial values. The numbers by the bars depict the number of cells analyzed. Data are shown as the means ± s.e.m. **(D,E)** Representative (above) and mean time-course (below) of the AKAR2 FRET ratio signal upon addition of **(D)** L-lactate (20 mM) and **(E)** 3Cl-5OH-BA (0.5 mM). Data are expressed as percentages of the FRET ratio signals (YFP/CFP) relative to the baseline ratio values. Single exponential rise functions were fitted to the curves: (**A**, above) *FRET ratio* = [1.01 ± 0.00] + [0.10 ± 0.00] × (1 – exp(–*t*/[90.91 ± 8.26 s])), (**A**, below) *FRET ratio* = [1.00 ± 0.00] + [0.08 ± 0.00] × (1 – exp(–*t*/[111.11 ± 9.91 s])), and (**B**, above) *FRET ratio* = [0.97 ± 0.01] + [0.10 ± 0.01] × (1 – exp(–*t*/[47.62 ± 4.54 s])), (**B**, below) *FRET ratio* = [0.98 ± 0.00] + [0.12 ± 0.00] × (1 – exp(–*t*/[71.43 ± 5.10 s])). Note that the addition of L-lactate and 3Cl-5OH-BA increased the FRET ratios, indicating increased PKA activity. Each data point represents the mean ± s.e.m. **(F)** Mean changes in the FRET ratio (ΔFRET ratio) and mean time-constants (τ) upon L-lactate and 3Cl-5OH-BA stimulation. The numbers by the bars depict the number of cells analyzed. Data shown are in the format of the mean ± s.e.m. Data for every set of experiment was acquired from at least two different animals.

**FIGURE 2:**
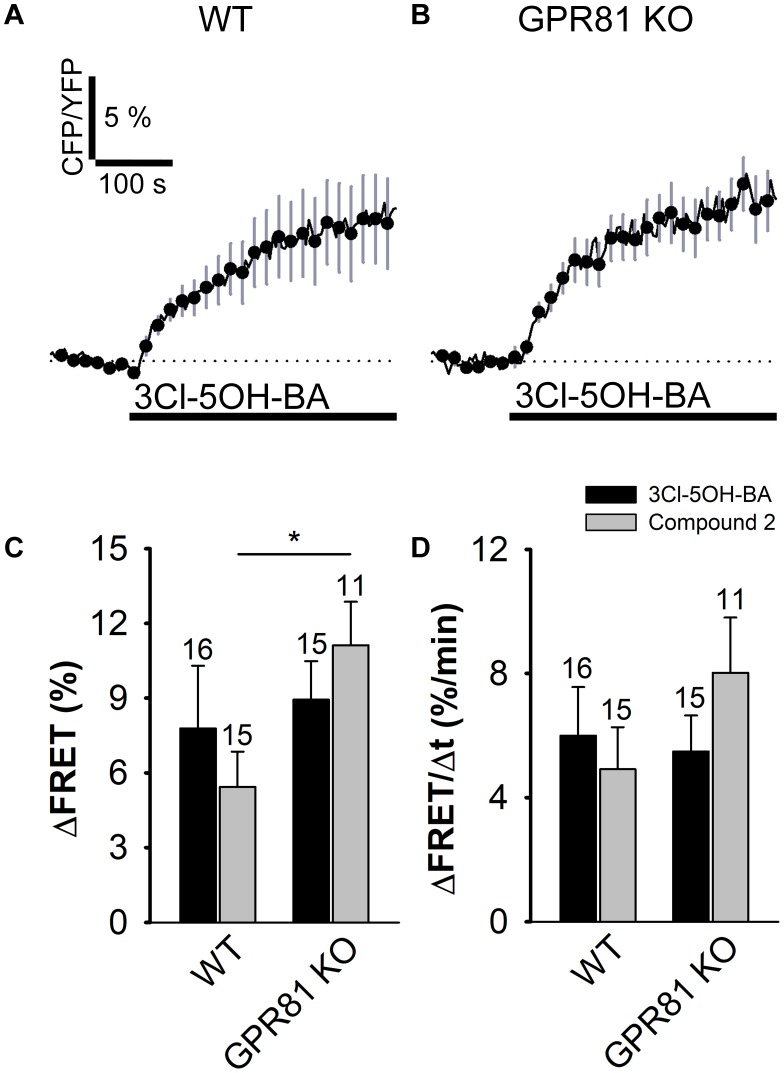
Stimulation with 3Cl-5OH-BA and Compound 2 elicits persistent increase in [cAMP]_i_ in WT and GPR81 KO mice astrocytes expressing the FRET nanosensor Epac1-camps. **(A,B)** Mean normalized time-courses of the Epac1-camps FRET ratio signal after stimulation with 3Cl-5OH-BA (0.5 mM) in **(A)** WT (*n* = 16) and **(B)** GPR81 KO (*n* = 15) cultured mouse astrocytes at *t* = 100 s (black lines). Changes in the FRET ratio signal are expressed as percentages of the inverse FRET ratio signal (CFP/YFP) relative to the baseline values. The monophasic exponential increase in the FRET signal (represented by the CFP/YFP ratio) after 3Cl-5OH-BA stimulation reflects an increase in [cAMP]_i_. Each data point represents mean ± s.e.m. **(C,D)** Mean changes in the Epac1-camps FRET ratio signal (ΔFRET; **C**) and initial rates of the FRET ratio signal increase (ΔFRET/Δt; **D**) after the addition of 3Cl-5OH-BA (black bars) and Compound 2 (gray bars) in WT and GPR81 KO astrocytes. Numbers by the error bars depict the number of cells analyzed. (^∗^*P* < 0.05; Mann Whitney *U* test) Data for every set of experiment was acquired from at least two different animals except for Compound 2.

**FIGURE 3:**
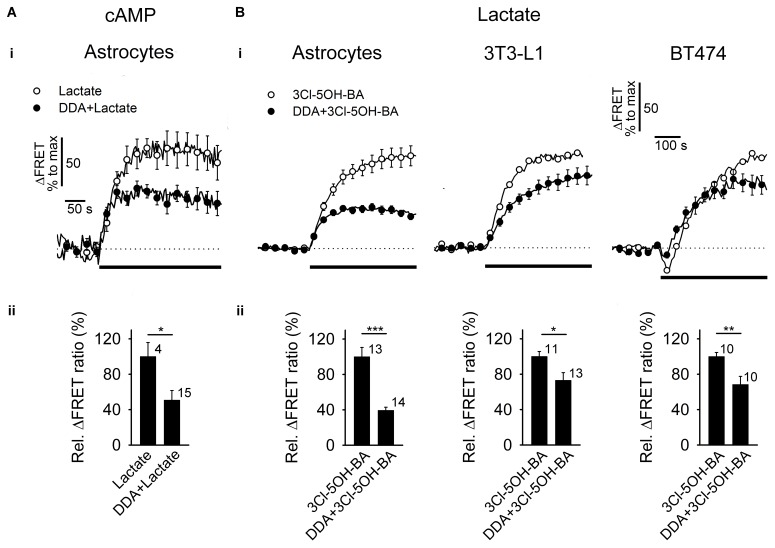
Inhibition of AC reduces the L-lactate- and 3Cl-5OH-BA-induced increase in [cAMP]_i_ and [lactate]_i_ **(A,B**, panels **i)** Mean time-courses of FRET ratio signal changes normalized to the maximum signal change for **(A,i)** Epac1-camps upon addition of 20 mM L-lactate and **(B,i)** Laconic upon addition of 0.5 mM 3Cl-5OH-BA (black lines) in the absence (white circles) and presence (black circles) of 100 μM DDA, an inhibitor of AC. Each data point represents mean ± s.e.m. (**A,B**, panels **ii)** Mean relative changes in FRET ratio (Rel. ΔFRET ratio) upon L-lactate **(A)** and **(B)** 3Cl-5OH-BA stimulation in the absence and presence of DDA. Relative ΔFRET values (%) were calculated by dividing individual ΔFRET values with the average ΔFRET value upon L-lactate or 3Cl-5OH-BA stimulation. Note that the inhibition of AC by DDA causes ∼50 % reduction in L-lactate-induced increase in [cAMP]_i_ in astrocytes and a ∼30–60% reduction in 3Cl-5OH-BA-induced increase in [lactate]_i_ in astrocytes, 3T3-L1 and BT474 cells. In BT474 cells the application of 3Cl-5OH-BA initiated a transient reduction in [lactate]_i_ that was diminished in the presence of DDA. Numbers adjacent to black bars represent number of cells. Data are in the format of the mean ± s.e.m (^∗^*P* < 0.05, ^∗∗^*P* < 0.01, ^∗∗∗^*P* < 0.001). Data for every set of experiment was acquired from at least two different animals.

We performed real-time monitoring of [cAMP]_i_ while extracellular L-lactate or the GPR81 lactate receptor agonist 3Cl-5OH-BA (Dvorak et al., 2012; Liu et al., 2012) were applied. Using the FRET-nanosensor for cAMP Epac1-camps, in 7 (37%) out of 19 cells an increase in [cAMP]_i_ was measured when a high concentration of L-lactate (20 mM) was applied (**Figure 1A**). Decreased [cAMP]_i_ was measured only in 3 (16%) cells out of 19 (**Table 1**). Although GPR81 receptor in adipocytes is coupled to G_i_-proteins decreasing [cAMP]_i_ (Ahmed et al., 2010), the observed L-lactate-mediated elevation in [cAMP]_i_ in 37% of cells studied might be due to the activation of a G_s_-protein coupled to L-lactate GPR81 receptor, as a similar rise in [cAMP]_i_ was recorded in astrocytes by adding the selective GPR81 receptor agonist 3Cl-5OH-BA (0.5 mM, **Figure 1B**); 9 (43%) of 21 cells responded with an increase (**Figure 1B**) and 2 (10%) with a small decrease in [cAMP]_i_ (**Table 1**). Inhibition of AC activity by 100 μM DDA (Vardjan et al., 2014) reduced the 20 mM extracellular L-lactate-induced increase in [cAMP]_i_ by ∼50 % (**Figure 3A**), indicating that in the majority of astrocytes, extracellular L-lactate activates the cAMP pathways via binding to receptors that activate AC.

To verify whether the 3Cl-5OH-BA-induced increase in [cAMP]_i_ is mediated via the GPR81 lactate receptor, we used isolated astrocytes from the GPR81 KO mice (Ahmed et al., 2010). Interestingly, even in GPR81 KO astrocytes, the application of 3Cl-5OH-BA (0.5 mM), like in WT astrocytes, elicited an increase in [cAMP]_i_ (**Figure 2** and **Table 1**). Similar results were obtained by using a much higher affinity GPR81 receptor agonist Compound 2 (Sakurai et al., 2014; histograms in **Figures 2C,D**; **Table 1**), suggesting that these agonists activate in astrocytes a second, yet unidentified receptor-like mechanism, thus resembling the unidentified L-lactate receptor in neurons, coupled to G_s_-protein increasing the production of cAMP (Tang et al., 2014).

To see if L-lactate and GPR81 agonist 3Cl-5OH-BA target the same, yet unidentified receptor-like mechanism, sharing the binding on the receptor, we pretreated rat astrocytes with the sub-effective dose of GPR81 receptor agonist 3Cl-5OH-BA (50 μM). The application of 50 μM 3Cl-5OH-BA is ineffective in increasing [cAMP]_i_ in astrocytes, since the application of 3Cl-5OH-BA results in a FRET ratio signal change (1.63 ± 0.38 %, *n* = 13), not significantly different to the vehicle-induced response in controls (1.01 ± 0.46 %, *n* = 13, *P* = 0.31). However, the sub-effective dose of 3Cl-5OH-BA (50 μM) reduced the L-lactate-induced elevation in [cAMP]_i_ (Supplementary Figure S4), indicating that in addition to interacting with the GPR81 lactate receptor, this supposedly selective GPR81 receptor agonist (Dvorak et al., 2012), binds like L-lactate to the yet unidentified receptor in astrocytes.

Furthermore, to evaluate whether the L-lactate receptor-like mechanism elevates [cAMP]_i_ and consequently also activates the cAMP-effector protein kinase A (PKA) we used the AKAR2, PKA activation nanosensor (Zhang et al., 2005). Significant increase in PKA activity was recorded (**Figures 1D,E**) when astrocytes were exposed to 20 mM L-lactate in 57% of 14 cells or to 0.5 mM 3Cl-5OH-BA in 56% of 16 cells (**Table 1**). The differences in the responsiveness of cells between Epac1-camps and AKAR2 nanosensors (∼40% vs. ∼60%, respectively) may be due to a higher sensitivity of the AKAR2 nanosensor, since Epac1-camps can only detect [cAMP]_i_ that is >100 nM (Börner et al., 2011). No changes in PKA activity were observed, when a 10-fold lower concentration of L-lactate (2 mM) was applied to cells (Supplementary Figure S5). The time-course and the extent of increase in the PKA activity were similar for both types of stimuli, L-lactate and 3Cl-5OH-BA (**Figure 1F**). We observed a delay between the addition of L-lactate or 3Cl-5OH-BA and subsequent increase in AKAR2 FRET ratio signal, determined from the intersection of the reference baseline with the exponential curve (time of delay was 35 ± 7 s (20 mM L-lactate) vs. 49 ± 7 s (0.5 mM 3Cl-5OH-BA); *P* = 0.19), consistent with PKA activation occurring downstream of cAMP production.

The observation that a 10-fold lower concentrations of 3Cl-5OH-BA (50 μM vs. 500 μM) and L-lactate (2 mM vs. 20 mM) does not affect cAMP levels and PKA activity in astrocytes, respectively, indicates that astrocytes respond to extracellular 3Cl-5OH-BA and L-lactate in a concentration-dependent manner, further suggesting that these two agonists in astrocytes cause a rise in cAMP via activation of an excitatory receptor-like mechanism in the plasma membrane.

### Extracellular L-Lactate and GPR81 Receptor Agonist Trigger Similar Increases in [lactate]_i_ in Astrocytes and Cancer Cells as Adrenergic Stimulation via Adenylate Cyclase Activation

Brain-based aerobic glycolysis (Goyal et al., 2014) is upregulated by noradrenergic stimuli affecting astrocyte metabolism (Dienel and Cruz, 2016). It has been reported that AR stimulation elevates astrocytic [cAMP]_i_ (Vardjan et al., 2014; Horvat et al., 2016) and also intracellular concentration in free glucose ([glucose]_i_; Prebil et al., 2011). We show here using lactate FRET nanosensor Laconic (San Martin et al., 2013) that astrocytes respond to AR activation also with an increase in intracellular L-lactate (Supplementary Figure S2) from its basal to the new steady state levels.

Although we have observed that in all studied astrocytes exposed to ISO (*n* = 8) and NA (*n* = 16) the [cAMP]_i_ increased, the elevations in [glucose]_i_ were only rarely observed, when cells were treated with selective β-AR agonist ISO [5 (16%) of the 31 cells]. The percentage of responsive cells, however, increased by twofold, when cells were exposed to NA, nonselective AR agonist [8 (40%) of the 20 cells; **Table 1**] and when cells were treated with selective α_1_-AR agonist (data not shown), implying the role of Ca^2+^ in the elevation of [glucose]_i_. In contrast to measurements of [glucose]_i_, the majority of astrocytes (63%) responded to ISO application with an elevation in [lactate]_i_ (10 out of 16 cells); 89% of astrocytes (8 out of 9 cells) responded with enhanced [lactate]_i_ to stimulation by NA (Supplementary Figure S2C, **Table 1**). The time-course of the measured [cAMP]_i_ increase was faster compared to that of [glucose]_i_ and [lactate]_i_ (Supplementary Figure S3).

**FIGURE 4:**
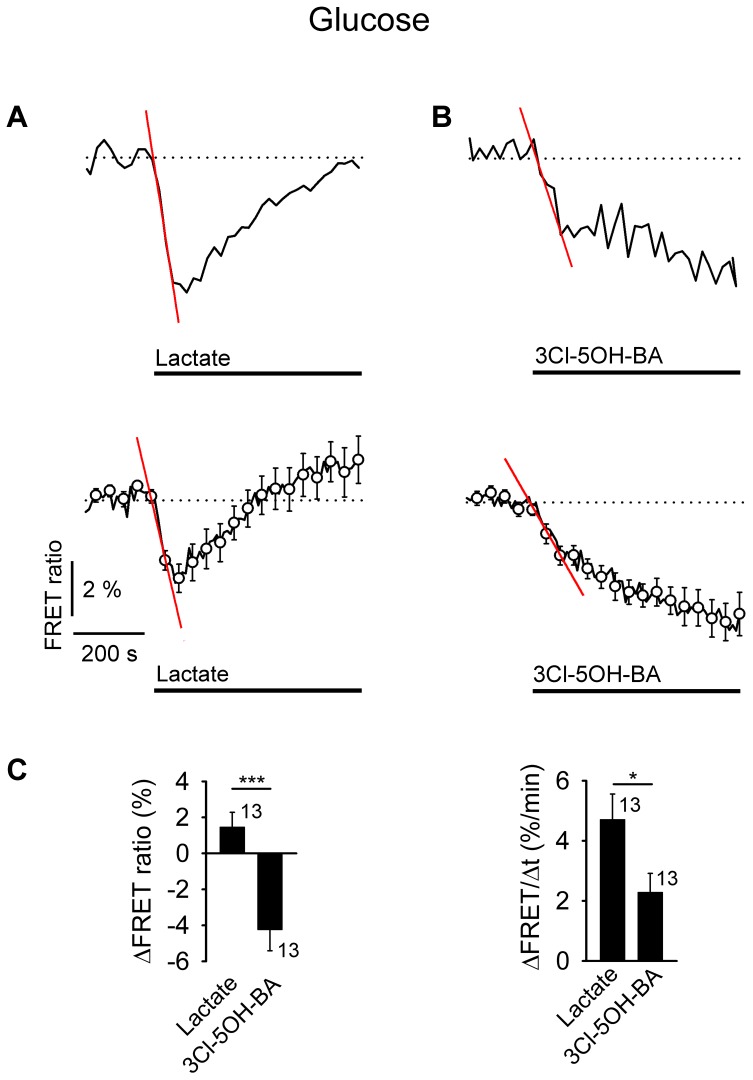
Application of L-lactate and 3Cl-5OH-BA decreases [glucose]_i_ in astrocytes. **(A,B)** Representative (above) and mean time-course (below) of FLII^12^Pglu-700 μδ6 FRET ratio signal, reporting [glucose]_i_, upon the addition of **(A)** L-lactate (20 mM) and **(B)** 3Cl-5OH-BA (0.5 mM). Data are expressed as the percentages of the FRET ratio signals (EYFP/ECFP) relative to the baseline ratio values. The initial rate of change in [glucose]_i_ was determined by fitting the regression lines to the signal decreases; ΔFRET (%) = –*k* (%/min) × Δ*t* (min), where *t* is the time and the *k*–slope is the initial ΔFRET decline: k_(A,above)_ = 4.94 ± 0.28 %/min, k_(A,below)_ = 2.90 ± 0.36 %/min, and k_(B,above)_ = 2.32 ± 0.29 %/min, k_(B,below)_ = 1.05 ± 0.11 %/min. Note that the addition of L-lactate transiently decreased the FRET ratio, while the 3Cl-5OH-BA persistently decreased the FRET ratio. Each data point represents the mean ± s.e.m. **(C)** Mean changes in the FRET ratio (ΔFRET ratio; left) and initial rates in the FRET ratio decrease (ΔFRET/Δ*t*; right) upon L-lactate and 3Cl-5OH-BA stimulation. Numbers adjacent to the bars depict the number of cells analyzed. Data shown are in the format of the mean ± s.e.m (^∗^*P* < 0.05, ^∗∗∗^*P* < 0.001). Data for every set of experiment was acquired from at least two different animals.

**FIGURE 5:**
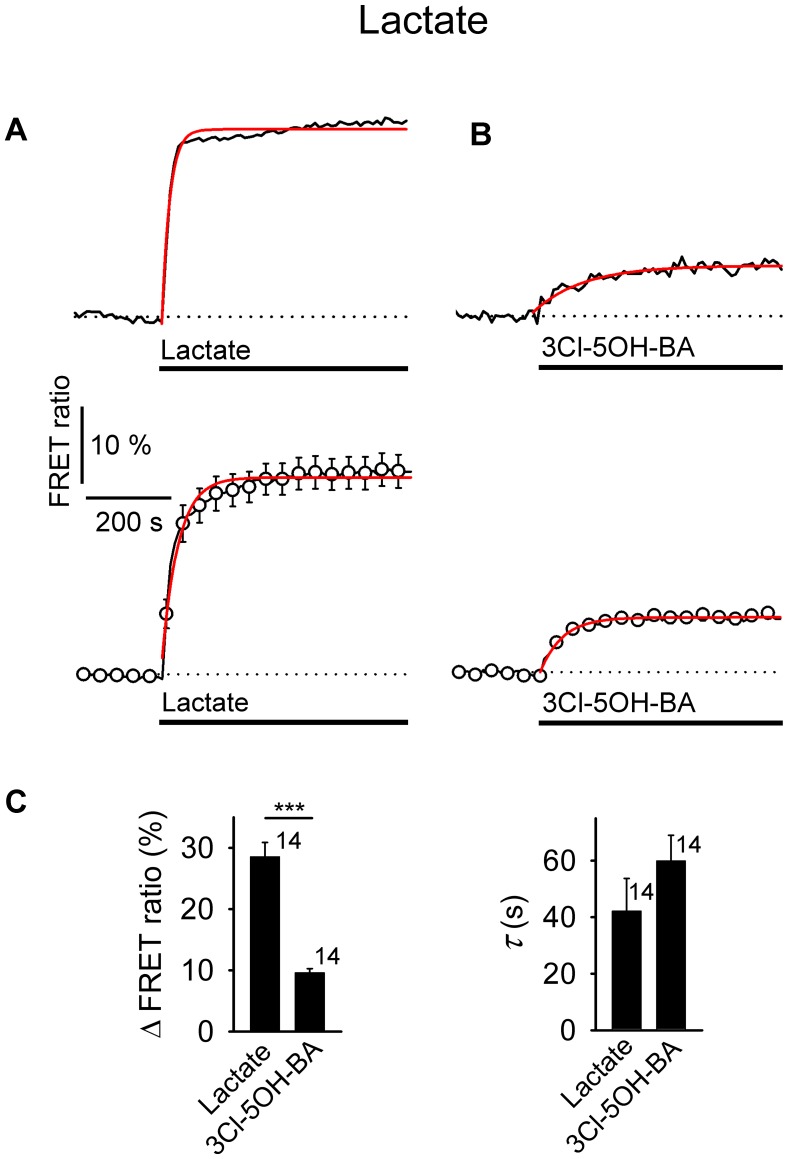
Comparison of increases in [lactate]_i_ by extracellular L-lactate and 3Cl-5OH-BA in astrocytes. **(A,B)** Representative (above) and mean time-course (below) of the Laconic FRET ratio signal upon the addition of **(A)** L-lactate (20 mM) and **(B)** 3Cl-5OH-BA (0.5 mM). Data are expressed as the percentages of FRET ratio signals (mTFP/Venus) relative to the baseline ratio values. Single exponential rise functions were fitted to the curves: (**A**, above) FRET ratio = [0.81 ± 0.02] + [0.44 ± 0.02] × (1 – exp (–*t*/[18.80 ± 1.10 s])), (**A**, below) FRET ratio = [1.02 ± 0.01] + [0.24 ± 0.01] × (1 – exp(–*t* / [39.06 ± 2.44 s])), and (**B**, above) FRET ratio = [1.00 ± 0.00] + [0.06 ± 0.00] × (1 – exp(–*t*/[116.28 ± 14.87 s])), (**B**, below) FRET ratio = [0.98 ± 0.00] + [0.09 ± 0.00] × (1 – exp(–*t*/[54.05 ± 2.34 s])). Note that the addition of L-lactate and 3Cl-5OH-BA increased the FRET ratios, indicating an increase in [lactate]_i_. Each data point represents the mean ± s.e.m. **(C)** Mean changes in the FRET ratio (ΔFRET ratio; left) and mean time-constants (τ; right) upon L-lactate and 3Cl-5OH-BA stimulation. Numbers by the error bars depict the number of cells analyzed. Data shown are in the format of the mean ± s.e.m (^∗∗∗^*P* < 0.001). Data for every set of experiment was acquired from at least two different animals.

Since L-lactate and GPR81 agonist 3Cl-5OH-BA (**Figure 1**), like β-AR agonists (Supplementary Figure S2; Vardjan et al., 2014; Horvat et al., 2016) increase [cAMP]_i_ and PKA activity in astrocytes, we further examined whether extracellular L-lactate or the GPR81 agonist 3Cl-5OH-BA affect [glucose]_i_ and [lactate]_i_ in astrocytes. **Figure 4** shows that both extracellular L-lactate (20 mM) and 3Cl-5OH-BA (0.5 mM) decreased [glucose]_i_ in the majority of studied cells. In the former case, the decrease was transient (65% of 20 cells responded), but the change was persistent in the latter case, where 68% of 19 cells responded. Interestingly, these treatments elevated [lactate]_i_ in 93% of 15 cells (L-lactate) and in all 14 (100%) cells in case of 3Cl-5OH-BA (**Figures 3B** (left panel), **5**). The increase in [lactate]_i_ was faster when the cells were exposed to extracellular L-lactate than 3Cl-5OH-BA, likely due to the entry of L-lactate into the cytosol through the plasma membrane MCTs. The extent of [lactate]_i_ elevation was more than twofold higher upon the addition of L-lactate vs. 3Cl-5OH-BA (**Figure 5C**), which was expected due to L-lactate entry into the cytoplasm via the MCTs and possibly channels (Sotelo-Hitschfeld et al., 2015).

A few cells responded with a [glucose]_i_ increase to L-lactate or 3Cl-5OH-BA stimulation [3 (15%) and 1 (5%) cells, respectively], but no cell responded with a decrease in [lactate]_i_ to these stimuli.

As in the case of the cAMP-dependent PKA activity (*n* = 7), the addition of 2 mM L-lactate had no significant effect on [glucose]_i_ (*n* = 11; Supplementary Figure S5), further indicating that astrocytes respond to extracellular L-lactate in a concentration-dependent manner.

Taken together these results show that in astrocytes, extracellular L-lactate and 3Cl-5OH-BA trigger an elevation in [lactate]_i_, likely via the cAMP-mediated activation of glucose consumption, as decreased [glucose]_i_ was recorded in the majority of the 3Cl-5OH-BA-treated cells. Moreover, inhibition of AC by 100 μM DDA (Vardjan et al., 2014), reduced the 3Cl-5OH-BA-induced increase in [lactate]_i_ in astrocytes. We have observed inhibition of 3Cl-5OH-BA-induced increase in [lactate]_i_ with DDA also in 3T3-L1 embryonic murine cells with genetic trait as seen in human ectodermal cancers (Leibiger et al., 2013) and in human mammary ductal carcinoma BT474 cells (**Figure 3B**). Both cell lines were shown to be positive for the GPR81 receptor (Liu et al., 2009; Staubert et al., 2015), indicating that AC activation by a membrane receptor is responsible for the increase in [lactate]_i_ in cells that exhibit aerobic glycolysis such as astrocytes and cancer cells (Supplementary Table S1).

### Receptor-Regulated Increase in the Rate of Aerobic Glycolysis in Astrocytes

**FIGURE 6:**
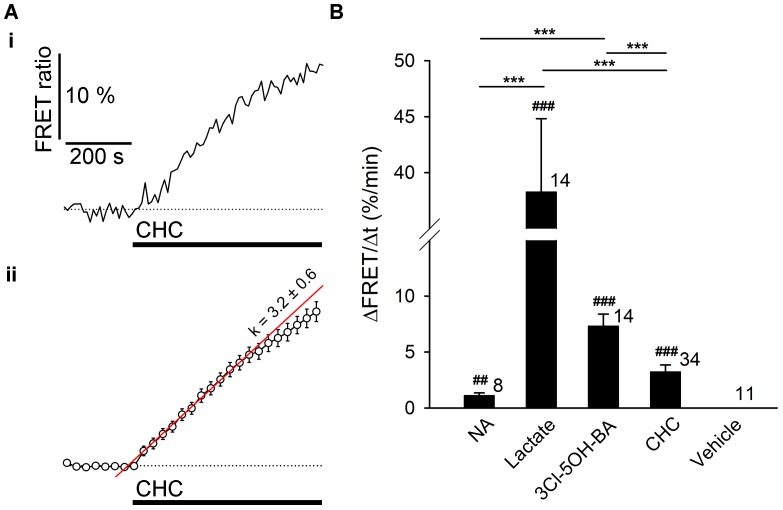
The resting rate of glycolysis and lactate production is modulated by noradrenaline and 3Cl-5OH-BA in astrocytes. **(A)** Representative **(i)** and mean **(ii)** time-course of Laconic FRET ratio signal upon addition of 6 mM CHC. Data are expressed as the percentage of the FRET ratio signal (mTFP/Venus) relative to the baseline ratio values. Note that the increased FRET ratio indicates an increase in [lactate]_i_. Each data point in **(ii)** represents mean ± s.e.m. The initial rate of change in [lactate]_i_ was determined by fitting the regression lines to the initial rise of the curves. The regression line in **(ii)** has the form of:ΔFRET (%) = *k* (%/min) × Δ*t* (min) + ΔFRET_0_, where *t* is time, ΔFRET_0_ is ΔFRET at the time of stimulus, and the *k*-slope is the initial ΔFRET rise: *k* = 3.2 ± 0.6 %/min. **(B)** Comparison of mean maximal initial rates in FRET ratio changes (ΔFRET/Δ*t*) upon the addition of NA (200 μM), L-lactate (20 mM), 3Cl-5OH-BA (0.5 mM), CHC (6 mM), and vehicle (control) in astrocytes. Maximal initial rates are expressed as percent change of FRET ratio per minute. Numbers by the error bars depict the number of cells analyzed. ^∗∗∗^*P* < 0.001 one-way ANOVA comparison between different types of stimuli; ^##^*P* < 0.01, ^###^*P* < 0.001 one-sample *t*-test. Data for every set of experiment was acquired from at least two different animals.

Aerobic glycolysis in the brain is likely mediated by astrocytes, since these cells strongly favor L-lactate as the end glycolytic product whether or not oxygen is present (Halim et al., 2010). To measure the glycolytic rate in these cells, we estimated the maximal initial rate of [lactate]_i_ increase in the presence of CHC (6 mM), a non-specific inhibitor of membrane MCTs (MCTs 1-4). In the absence of L-lactate exchange through the plasma membrane, the predominant pathway available for cytosolic L-lactate accumulation is considered to be glycolytic L-lactate production. Although cytosolic L-lactate may be transported to mitochondria for oxidation in astrocytes (Passarella et al., 2008), in the presence of MCT blockers the initial rate of cytosolic L-lactate accumulation, measured as ΔFRET/Δt, is comparable to that of the initial rate of aerobic glycolysis. **Figure 6A**, ii shows that upon the addition of CHC, the initial maximal rate of the [lactate]_i_ increase was 3.2 ± 0.6 %/min (*P* < 0.001), which was consistent with previous results (Sotelo-Hitschfeld et al., 2015). It appears that this effect is not an artifact, as vehicle addition did not affect the [lactate]_i_ (**Figure 6B**; *P* = 0.18). With the addition of 3Cl-5OH-BA (0.5 mM), the rate of [lactate]_i_ increase was approximately twofold higher than the resting rate of glycolysis. NA (200 μM) appeared as a weaker stimulus of [lactate]_i_ increase in comparison to 3Cl-5OH-BA (0.5 mM) (**Figure 6B**) at the concentrations of respective agonists used. The strongest effect on the measured rate in the [lactate]_i_ increase was recorded when extracellular L-lactate (20 mM) was applied. This was expected, as L-lactate can enter the cytoplasm via the MCTs. Consistent with this finding, when L-lactate was added in the presence of the MCT blocker CHC, the increase in [lactate]_i_ was strongly attenuated in comparison to the conditions in the absence of CHC (data not shown).

Taken together, the results indicate that in astrocytes, extracellular L-lactate mediates an increase in [cAMP]_i_ that stimulates aerobic glycolysis and elevates [lactate]_i_ via a receptor-mediated pathway involving AC.

## Discussion

Here we studied whether extracellular L-lactate affects aerobic glycolysis via G-protein coupled L-lactate receptors, such as the GPR81 receptor in astrocytes (Lauritzen et al., 2014; Sharma et al., 2015). The main finding of this study is that extracellular L-lactate in astrocytes activates AC, elevates [cAMP]_i_, and accelerates aerobic glycolysis. Interestingly, by using the selective agonists for the GPR81 receptor, such as 3Cl-5OH-BA (Dvorak et al., 2012; Liu et al., 2012) or Compound 2 (Sakurai et al., 2014), the results revealed that even in the absence of the GPR81 receptor expression in astrocytes from GPR81 KO mice, elevations in [cAMP]_i_ were still recorded, indicating that in addition to the GPR81 receptors, these agonists activate also a yet unidentified L-lactate receptor-like mechanism.

### L-Lactate and Adrenergic Receptor Stimulation Increases [lactate]_i_ in Astrocytes

Genes associated with aerobic glycolysis are mainly expressed in neocortical areas where neuronal cell plasticity is taking place (Goyal et al., 2014). In these areas, astrocytes are considered the primary site of aerobic glycolysis, and the primary source of L-lactate release. The results of this study demonstrate that the activation of astrocytic receptors by extracellular L-lactate (20 mM) or the GPR81 lactate receptor agonist 3Cl-5OH-BA (0.5 mM) (Dvorak et al., 2012) increases [lactate]_i_ within 1 min to a relatively high and stable level.

**FIGURE 7:**
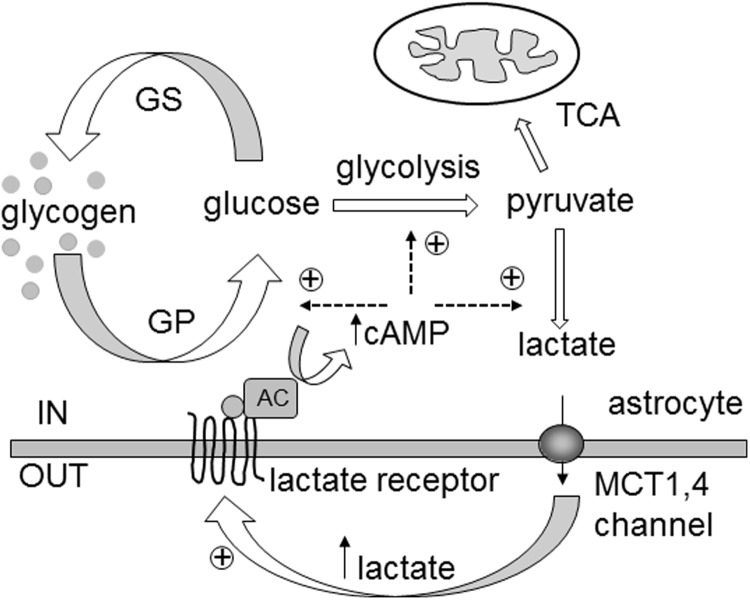
Extracellular L-lactate controls cytosolic lactate synthesis via yet unidentified receptors coupled to adenylate cyclase activity and cytosolic cAMP increase in astrocytes. In the brain, L-lactate is formed in the cytoplasm of astrocytes (IN) and is released through monocarboxylate transporters (MCTs) 1,4 and/or lactate-permeable channels. Extracellularly (OUT), L-lactate can be transported to neighboring cells as a fuel. However, it can also act as a signaling molecule by binding to the L-lactate receptors of neighboring cells, stimulating adenylate cyclase activity (AC) and an increase in cAMP synthesis. Elevated cytosolic cAMP levels facilitate glycogen degradation by activating glycogen phosphorylase (GP) and glycolysis with L-lactate as the end product. In the absence of the L-lactate positive feedback mechanism (“metabolic excitability”), the L-lactate tissue concentration gradient could be dissipated, reducing the availability of L-lactate as a metabolic fuel, when local energy demands, especially in the brain, are high. This model shares similarities with the ‘autocrine lactate loop’ acting (oppositely) on [cAMP]_i_ through GPR81 receptor in adipocytes (Ahmed et al., 2010). GS, glycogen synthase; TCA, tricarboxylic acid cycle. Glucose denotes phosphorylated and free glucose.

If the L-lactate-mediated increase in [cAMP]_i_ is present *in vivo* it may contribute to the maintenance of the L-lactate gradient between astrocytes and neurons (Mächler et al., 2016) and also the gradient between the extracellular L-lactate in the brain and in the plasma (Abi-Saab et al., 2002). A positive feedback mechanism involving L-lactate as an extracellular signal that controls L-lactate production and subsequent L-lactate release from astrocytes (**Figure 7**) may be important during neuronal activity, when [lactate]_i_ may rapidly decline in astrocytes due to its facilitated exit through lactate MCTs and putative ion channels (Sotelo-Hitschfeld et al., 2015) and/or its diffusion through the gap junctions in astroglial syncytia (Hertz et al., 2014). Thus, in the absence of a positive feedback mechanism, the L-lactate concentration gradient may dissipate, reducing the availability of L-lactate as a metabolic fuel for neurons (Barros, 2013; Mosienko et al., 2015) and thereby limiting the support for neural network activity.

Experiments in slices have revealed that optical activation of astrocytes in LC triggers the release of L-lactate from astrocytes, which then excites LC neurons and triggers the widespread release of NA, likely in a receptor-dependent manner involving AC and PKA activation (Tang et al., 2014). It has been proposed that astrocytes also release L-lactate at the axonal varicosities of the noradrenergic neurons, where it facilitates the release of NA from the varicosities (Tang et al., 2014). However, whether NA released from the LC neurons affects astrocyte L-lactate production has not been studied directly.

The real-time [lactate]_i_ monitoring in this study revealed that AR activation by NA can elicit a sustained increase in [lactate]_i_ in astrocytes, with an average time constant of ∼100 s (Supplementary Figure S2). The increase in [lactate]_i_ is predominantly a consequence of increased L-lactate production in aerobic glycolysis, although changes in L-lactate fluxes across the plasma membrane or astrocytic consumption of L-lactate in oxidative metabolic pathway could also partly contribute to the new L-lactate steady state levels (San Martin et al., 2013).

The increased [lactate]_i_ likely leads to L-lactate exiting astrocytes, which, *in vivo*, may back-excite LC neurons and further stimulate NA release (Tang et al., 2014). Such bidirectional communication between astrocytes and neurons may be part of a tissue-coordinated astrocyte activity, which can be studied by monitoring widespread LC-mediated Ca^2+^ signaling in astrocytes in awake-behaving mice *in vivo* (Ding et al., 2013; Paukert et al., 2014). Consistent with this possibility, cultured primary cortical neurons respond to extracellular L-lactate and the GPR81 lactate receptor agonists with a decrease in spontaneous Ca^2+^-spiking activity that is concentration-dependent, with 50% inhibitory concentration (IC_50_) of ∼4 mM L-lactate (Bozzo et al., 2013), which is close to the sensitivity of the GPR81 receptor for L-lactate (Liu et al., 2012).

### Extracellular L-Lactate-Induced Increase in [lactate]_i_ Involves Receptor-Like Mediated Adenylate Cyclase Activation and cAMP Production

In astrocytes, similar to the activation of β-ARs (Vardjan et al., 2014; Horvat et al., 2016), extracellular L-lactate (20 mM) and GPR81 lactate receptor agonist 3Cl-5OH-BA (0.5 mM) elevate [cAMP]_i_ (**Figures 1A,B**), as independently confirmed by monitoring the increase in activity of cAMP effector PKA (**Figures 1D,E**), but do not elevate [Ca^2+^]_i_ (data not shown). Thus, extracellular L-lactate in astrocytes likely stimulates a plasma membrane L-lactate sensitive receptor, such as the recently identified GPR81 receptor in brain astrocytes, which was linked to downregulation of cAMP synthesis (Lauritzen et al., 2014). To exclude the possibility that the addition of extracellular L-lactate acidifies the cytoplasm, thus affecting the fluorescence of fluorophores in a FRET nanosensor, especially the fluorescence of YFP (Patterson et al., 2001), which declines with acidification, producing an artifact that can be read as an increase in cAMP, we used two different types of FRET nanosensors to monitor cAMP activity; Epac1-camps (Nikolaev et al., 2004), and AKAR2 (Zhang et al., 2005), which report cAMP activity in opposite directions, as decrease and increase in YFP/CFP ratio, respectively. While in Epac1-camps transfected cells the L-lactate application induced a reduction in the YFP/CFP ratio, AKAR2-transfected cells responded to L-lactate with the increase in YFP/CFP ratio. Since the readouts from the Epac1-camps and AKAR2 YFP/CFP signals are in opposite directions it is highly unlikely that the observed YFP/CFP responses are due to acidification of the cytoplasm and not a consequence of cAMP activity. Furthermore, if the responses in cAMP and PKA activity would simply be due to a pH-dependent artifact, then we would have recorded signals that would be in phase with the application of L-lactate. This was not the case. As the PKA response was significantly delayed, it is more likely that delayed PKA activity is due to an elevation of cAMP, followed by the cAMP-mediated activation of PKA.

Interestingly, in both mouse WT and GPR81 KO astrocytes an increase in [cAMP]_i_ was detected (**Table 1**), suggesting that the observed effects of L-lactate and 3Cl-5OH-BA on cAMP signaling and metabolism in astrocytes are GPR81 independent. These results indicate the existence of a yet unidentified receptor-like mechanism of L-lactate production, activated with L-lactate as well as with the GPR81 selective agonists, 3Cl-5OH-BA (**Figure 1**) and Compound 2 (**Figure 2**; Sakurai et al., 2014). The unidentified receptor-like mechanism exhibits a relatively low 3Cl-5OH-BA affinity (we could not observe any response using 50 μM concentration) in comparison with the GPR81 lactate receptor (EC_50_ = 17 μM for 3Cl-5OH-BA) (Dvorak et al., 2012). Pretreatment of rat astrocytes with the 50 μM (subeffective) dose of 3Cl-5OH-BA reduced the L-lactate-induced elevations in [cAMP]_i_, indicating that this GPR81 receptor agonist may share with L-lactate the binding site on a yet unidentified receptor stimulating L-lactate production in astrocytes.

The synthesis of cAMP upon receptor activation is likely achieved via activation of AC, as was suggested for LC neurons, where extracellular L-lactate has been considered to activate AC and PKA, although the receptor triggering cAMP elevation in LC neurons is unknown (Tang et al., 2014; Mosienko et al., 2015). The application of DDA, an AC inhibitor, reduced the L-lactate-mediated increase in [cAMP]_i_ and also the 3Cl-5OH-BA-induced increase in [lactate]_i_ (**Figure 3**). An AC-dependent activity by the 3Cl-5OH-BA-induced increase in [lactate]_i_ appears to be taking place also in 3T3-L1 and BT474 cells, indicating that the Warburg effect-bearing cells regulate L-lactate synthesis via receptors (Supplementary Table S1).

A 10-fold lower L-lactate concentration (2 mM) did not affect [cAMP]_i_ or [glucose]_i_, implying a role of L-lactate receptor-like mechanism *in vivo* only at relatively elevated extracellular L-lactate levels, as likely takes place during exercise (Matsui et al., 2017). During abnormal conditions (e.g., hypoxia, hyperglycemia, seizures) the local resting extracellular L-lactate concentration of 0.1–2 mM in the narrow brain interstices (in humans 5 mM; Abi-Saab et al., 2002) can increase ∼10- to 20-fold to values >10 mM (Barros, 2013; Mosienko et al., 2015). Moreover, L-lactate production in the normal brain might occur in microdomains, which could create higher-than-average local concentrations (Morland et al., 2015; Mosienko et al., 2015).

In a few rat astrocytes (up to 16%, **Table 1**), the addition of 3Cl-5OH-BA, but not AR agonists, resulted in small sustained decreases in [cAMP]_i_ and PKA activity (**Table 1**), indicating that in these cells, L-lactate preferentially activates receptors coupled to the G_i_-proteins to downregulate cAMP, i.e., the coupling originally identified for GPR81 receptor (Ahmed et al., 2010). In mouse WT and GPR81 KO astrocytes, however, the decrease in [cAMP]_i_ was not detected (**Table 1**). The observed heterogeneity in the recorded L-lactate receptor-mediated cAMP responses may be species specific and/or due to molecular and functional heterogeneity of the astrocytes (Zhang and Barres, 2010), determined by the neuron-specific circuits (Chai et al., 2017).

The time-constants of the increases in [cAMP]_i_ and [lactate]_i_ were similar (58 s vs. 60 s, respectively; *P* = 0.90) upon 3Cl-5OH-BA application. However, upon AR activation, the increase in [cAMP]_i_ was approximately fivefold faster than that for the [lactate]_i_ increase (20 s vs. 105 s, respectively; *P* < 0.001; Supplementary Figure S2). The observed difference could be due to the distinct molecular coupling mechanisms between the respective receptors and the cAMP pathway generating distinct cAMP pools inside cells (compartmentalized cAMP signaling; Pidoux and Taskén, 2010), but altogether, the results indicate that lactate production depends on cAMP and occurs downstream of cAMP synthesis.

Taken together, these results show that the activation of not only ARs but also L-lactate receptor-like mechanism can accelerate L-lactate production in astrocytes via cAMP signaling, suggesting the existence of a yet unidentified excitatory L-lactate receptor in astrocytes. These findings diverge from the previously reported results in CHO-K1 cells (Cai et al., 2008), primary cortical neurons (Bozzo et al., 2013), homogenized mouse adipose tissue slices (Ahmed et al., 2010), and rat hippocampal slices (Lauritzen et al., 2014). In these tissues L-lactate (presumably via activation of the L-lactate GPR81 receptor) was considered to inhibit cAMP and subsequent L-lactate production.

### L-Lactate Receptor-Like Mechanisms Increase Intracellular L-Lactate More Potently Than Adrenergic Receptors

In astrocytes the NA-mediated glycogen breakdown and [glucose]_i_ increase (Prebil et al., 2011) may regulate the extent of aerobic glycolysis, a metabolic process favored in astrocytes (Gandhi et al., 2009; Barros, 2013). Intracellular [glucose]_i_ is (i) a function of glucose uptake from the extracellular space, (ii) is affected by glycogen degradation (Prebil et al., 2011), and (iii) involves the activity of glucose-6-phosphatase, present in astrocytes (Ghosh et al., 2005; Sharma et al., 2015), which converts glucose-6-phosphate to free glucose.

Glycogenolysis in astroglial cells is considered to be mainly regulated by β-ARs, although α_2_-ARs may also enhance it (Subbarao and Hertz, 1990; Hertz et al., 2010). Consistent with this possibility, the responsiveness of astrocytes to β-AR stimulation was lower than to α-/β-AR agonist NA when [lactate]_i_ was measured [63% (ISO) and 90% (NA), respectively, **Table 1**]. Upon the addition of 3Cl-5OH-BA, in contrast to adrenergic stimulation, a sustained decrease in [glucose]_i_ was recorded, indicating that D-glucose is rapidly consumed upon 3Cl-5OH-BA-mediated L-lactate receptor-like mechanism of activation (**Figure 4**). However, extracellular L-lactate triggered only a transient decrease in D-glucose, likely due to its interference with cytoplasmic L-lactate-sensitive enzymes (Costa Leite et al., 2007).

To estimate the extent by which the ARs and L-lactate receptors modulate the rate of aerobic glycolysis, we monitored [lactate]_i_ at rest in the presence of blocked L-lactate membrane transport. The addition of CHC, an inhibitor of several MCTs, resulted in a persistent increase in [lactate]_i_ (**Figure 6**). If it is assumed that L-lactate cannot exit cells under these conditions, the putative L-lactate-permeable channels are inactive (Sotelo-Hitschfeld et al., 2015), and L-lactate is not substantially metabolized (Passarella et al., 2008) then the rate of change in [lactate]_i_ reflects the resting rate of aerobic glycolysis in astrocytes. The addition of NA accelerated the rate of glycolysis to only ∼30% of the resting rate, whereas in the presence of 3Cl-5OH-BA, the glycolytic rate was increased by >600% relative to the NA-induced response (**Figure 6**). Although the effectiveness of the agonists used and their affinities for respective receptors may not be easily compared, these results suggest that L-lactate via receptor-like mechanism activates glycolysis more potently vs. ARs in astrocytes.

The results of this work bring new insights that in astrocytes L-lactate receptor-like mechanisms increase the rate of aerobic glycolysis, which manifests itself in increased [lactate]_i_. This metabolite can exit astrocytes to further accelerate L-lactate signaling at autocrine and paracrine sites. Hence, designating this process as “metabolic excitability” appears appropriate, as it may provide the means for maintaining a high and stable source of L-lactate levels in astrocytes, as measured *in vivo* (Mächler et al., 2016) and likely contributes to the difference between the brain extracellular fluid and plasma levels of L-lactate (Abi-Saab et al., 2002).

## Author Contributions

NV conceived and co-directed the study, performed experiments, analyzed data, and wrote the manuscript. HHC, AH, JV, MMu, STB, KM, and SP performed experiments and analyzed data. MMa and ŠGK performed experiments. MK analyzed data. GH designed and provided custom synthesized Compound 2. LHB and JS-M provided GPR81 KO mouse pups (founder mice from SO’s lab). SO generated the GPR81 KO mouse line. RZ conceived and directed the study and wrote the manuscript. All authors read and contributed to the completion of the draft manuscript.

## Conflict of Interest Statement

The authors declare that the research was conducted in the absence of any commercial or financial relationships that could be construed as a potential conflict of interest.

## Footnotes

1 www.addgene.org
