# Supplementary material for: Enhancement of Astroglial Aerobic Glycolysis by Extracellular Lactate-Mediated Increase in cAMP
Source: Front Mol Neurosci. 2018 May 8;11:148. doi: 10.3389/fnmol.2018.00148 (PMC5953330; doi:10.3389/fnmol.2018.00148)
Supplement: Supplementary file 1 [file Table_1.DOCX]

**Supplementary Table**

## Table S1: Responsiveness of cells to L-lactate- and 3Cl-5OH-BA-induced changes in intracellular L-lactate and cAMP in the presence and absence of adenylate cyclase inhibitor DDA.

| **Cell type** | **Stimulus** | ***n* (%)**  **increase** | ***n* (%)**  **transient**  **increase** | ***n* (%)**  **decrease** | ***n* (%)**  **unresponsive** | ***n***  **all** |
| --- | --- | --- | --- | --- | --- | --- |
|  | *Laconic* |  |  |  |  |  |
| **3T3-L1** | 3Cl-5OH-BA (0.5 mM) | 11 (100%) | 0 (0%) | 0 (0%) | 0 (0%) | 11 |
|  | DDA (100 μM)+3Cl-5OH-BA (0.5 mM) | 13 (92.8%) | 0 (0%) | 0 (0%) | 1 (7%) | 14 |
| **BT474** | 3Cl-5OH-BA (0.5 mM) | 10 (62.5%) | 0 (0%) | 1 (6.2%) | 5 (31.2%) | 16 |
|  | DDA (100 μM)+3Cl-5OH-BA (0.5 mM) | 10 (100%) | 0 (0%) | 0 (0%) | 0 (0%) | 10 |
| **Astrocytes** | 3Cl-5OH-BA (0.5 mM) | 13 (100%) | 0 (0%) | 0 (0%) | 0 (0%) | 13 |
|  | DDA (100 μM)+3Cl-5OH-BA (0.5 mM) | 14 (100%) | 0 (0%) | 0 (0%) | 0 (0%) | 14 |
|  | *Epac1-camps* |  |  |  |  |  |
| **Astrocytes** | L-lactate (20 mM) | 4 (100%) | 0 (0%) | 0(20%) | 0 (0%) | 4 |
|  | DDA (100 μM)+L-lactate (20 mM) | 9 (60%) | 4 (26.7%) | 0 (0%) | 2 (13.3%) | 15 |

3Cl-5OH-BA, 3-chloro-5-hydroxybenzoic acid; DDA, 2',3' dideoxyadenosine (adenylate cyclase inhibitor); *n*, number of cells; Laconic, lactate FRET-nanosensor; Epac1-camps, cAMP FRET-nanosensor
