# Supplementary material for: Enhancement of Astroglial Aerobic Glycolysis by Extracellular Lactate-Mediated Increase in cAMP
Source: Front Mol Neurosci. 2018 May 8;11:148. doi: 10.3389/fnmol.2018.00148 (PMC5953330; doi:10.3389/fnmol.2018.00148)
Supplement: Supplementary file 2 [file Data_Sheet_1.docx]

**Supplementary Material**

**Supplementary Figures and Figure Legends**

**
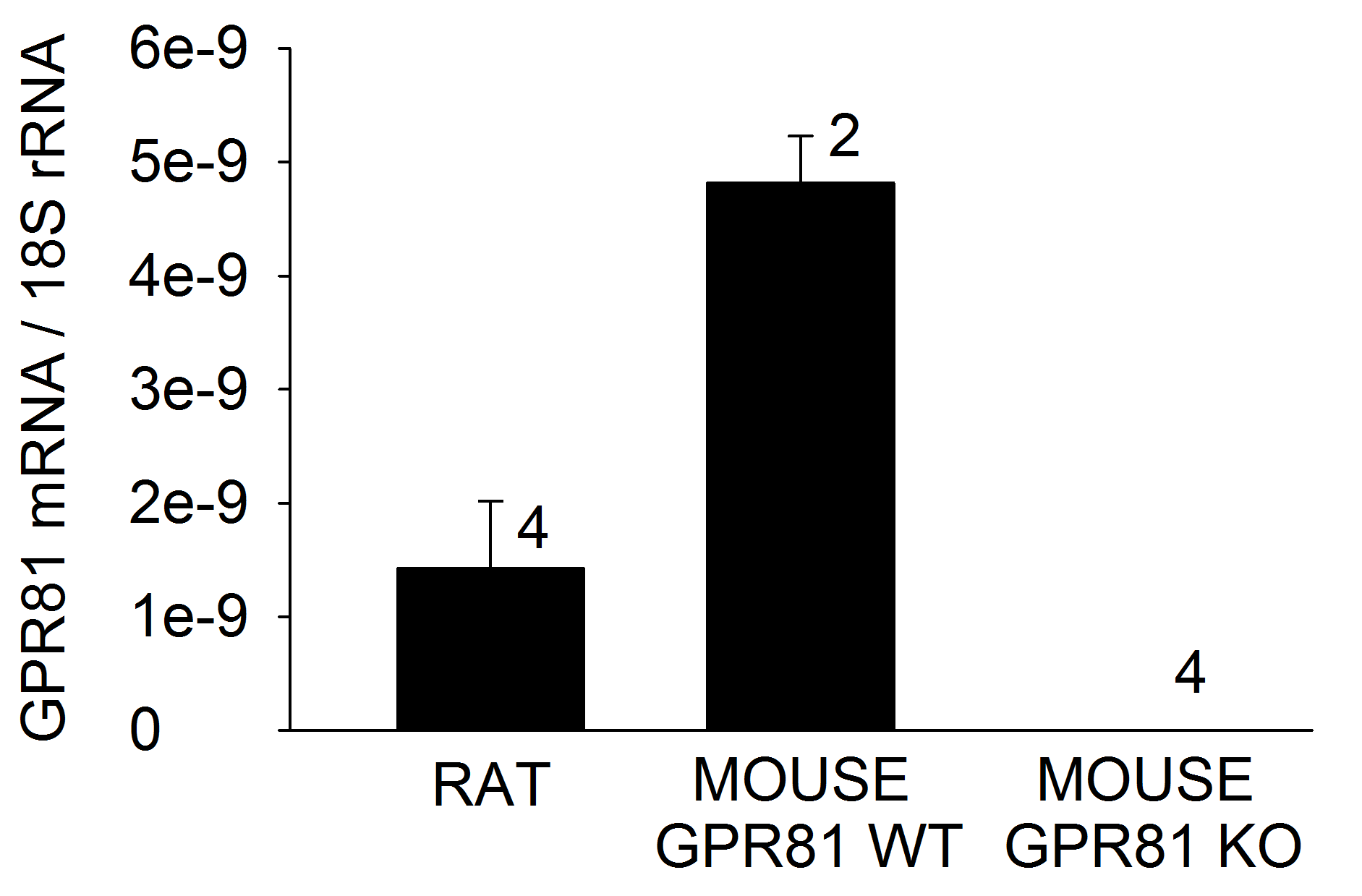
**

## Figure S1 Expression of GPR81 mRNA in cultured astrocytes.

GPR81 mRNA was measured in rat, WT mouse and GPR81 KO mouse astrocytes using qPCR. Expression level of GPR81 mRNA was normalized to the expression of 18S rRNA. Data are in the format means ± s.e.m. Numbers adjacent to columns represent different cell preparations.


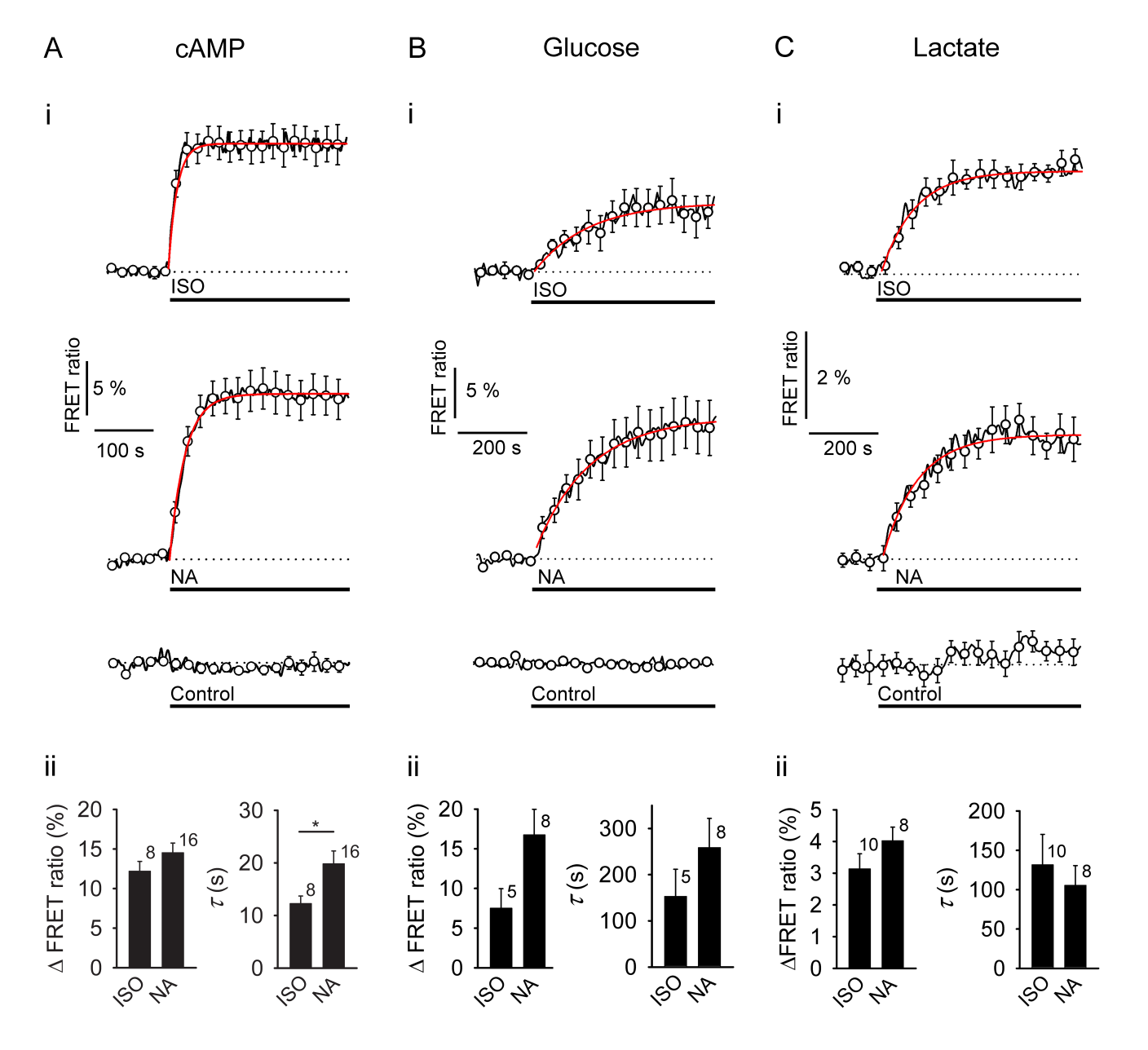


## Figure S2 Adrenergic stimulation increases [cAMP]_i_, [glucose]_i_, and [lactate]_i_ in cultured rat astrocytes

(A-C, panels i) Mean time-courses of (A) Epac1-camps, reporting cAMP levels (cAMP) (B) FLII^12^Pglu-700µδ6, reporting glucose levels (Glucose) and (C) Laconic FRET ratio signal, reporting cytosolic levels of L-Lactate (Lactate) upon addition of ISO (10-200 µM), NA (1-200 μM), and extracellular solution (Control). Data are expressed as the FRET ratio signals normalized to the baseline ratio values: the CFP/YFP ratio for cAMP, the EYFP/ECFP ratio for D-glucose and the mTFP/Venus ratio for L-lactate. Changes in the FRET ratio are expressed as percentages relative to the initial values. Single exponential rise functions were fitted to the curves: (A) *FRET ratio* = [0.99 ± 0.00] + [0.13 ± 0.00] × (1 – exp(–*t* / [12.50 ± 0.63 s]) for ISO and *FRET ratio* = [0.99 ± 0.00] + [0.17 ± 0.00] × (1 – exp(–*t* / [25.64 ± 0.66 s]) for NA, (B) *FRET ratio* = [1.00 ± 0.00] + [0.07 ± 0.00] × (1 – exp(–*t* / [166.67 ± 20.30 s]) for ISO and *FRET ratio* = [1.01 ± 0.00] + [0.13 ± 0.00] × (1 – exp(–*t* / [166.67 ± 8.71 s]) for NA, (C) *FRET ratio* = [1.00 ± 0.00] + [0.03 ± 0.00] × (1 – exp(–*t* / [98.04 ± 6.73s]) for ISO and *FRET ratio* = [1.00 ± 0.00] + [0.03 ± 0.00] × (1 – exp(–*t* / [103.09 ± 8.50 s]) for NA. Note that the addition of NA and ISO increased the FRET ratios, indicating an increase in the [cAMP]_i_, [glucose]_i_, and [lactate]_i_. Each data point represents the mean ± s.e.m. (A-C, panels ii) Mean changes in the FRET ratio (ΔFRET ratio) and mean time-constants (τ) upon NA and ISO stimulation separately for (A) cAMP, (B) glucose and (C) lactate sensors. The numbers by the error bars depict the number of cells analyzed. Data shown are in the format mean ± s.e.m (**P* < 0.05) Data for every set of experiment was acquired from at least two different animals.


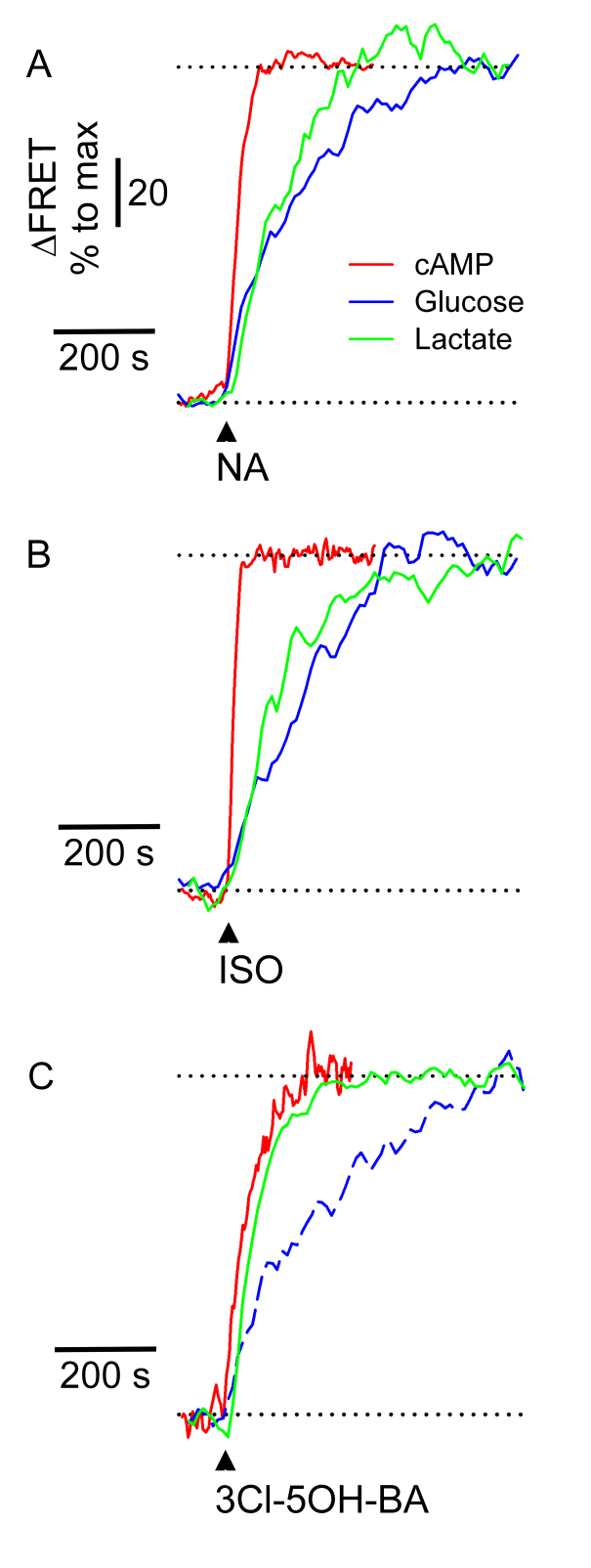


## Figure S3 Time-dependent changes in cytosolic cAMP, glucose and lactate upon adrenergic stimulation or addition of 3Cl-5OH-BA

(A-C) Mean time-course of FRET ratio signal changes normalized to the maximum signal change for Epac1-camps, FLII^12^Pglu-700µδ6, and Laconic FRET nanosensors, reporting changes in intracellular levels of cAMP (red line), glucose (blue line), and lactate (green line) upon the addition of noradrenaline (A; NA), isoprenaline (B; ISO), and 3Cl-5OH-BA (C). Note that solid and dashed lines reflect increases and decreases, respectively, in [cAMP]_i_, [glucose]_i_, or [lactate]_i_. Traces are from experiments shown in Figures S2, 1, 4 and 5.


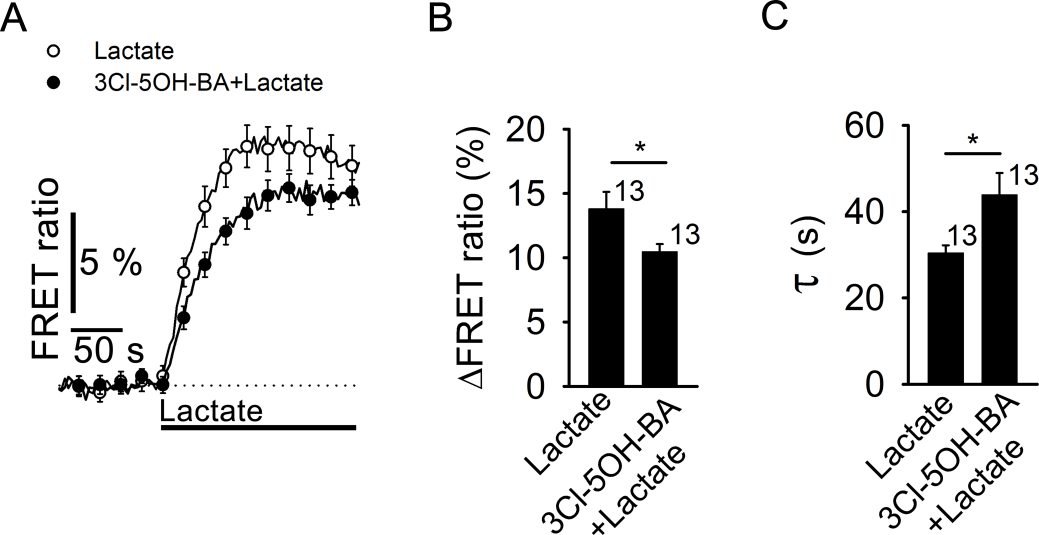


## Figure S4 Pretreatment of astrocytes with subeffective concentrations of 3Cl-5OH-BA reduces the L-lactate-induced increase in [cAMP]_i_

(A) Mean normalized time-courses of the Epac1-camps FRET ratio signal (CFP/YFP) upon the addition of L-lactate (20 mM) in control (open circles) and in cells pretreated with 3Cl-5OH-BA (50 µM, full circles), a selective agonist of the GPR81 receptor. Changes in the FRET ratio signal are expressed as percentages relative to the baseline values. (B) Mean maximal changes in the FRET ratio (ΔFRET ratio) and (C) mean time-constants (τ) upon L-lactate stimulation in control cells and in cells pretreated with 3Cl-5OH-BA. ΔFRET ratio and τ values were determined from the exponential rise to maximum functions that were fitted to the FRET curves as soon as they reached maximal values. Numbers by the error bars depict the number of cells analysed. Data are presented as means ± s.e.m. (**P* < 0.05). Data for every set of experiment was acquired from at least two different animals.

**
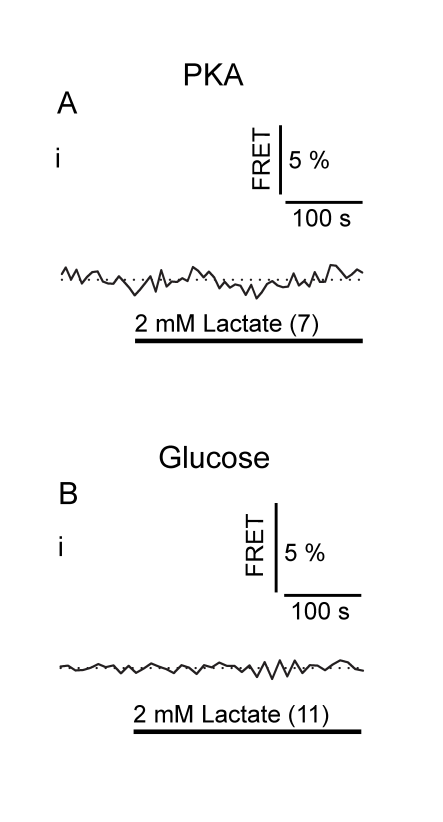
**

## Figure S5 Effects of 2 mM L-lactate on cAMP-dependent PKA activity and [glucose]i in astrocytes

(A-B) Mean time-course of FRET ratio signal changes for (A) AKAR2 and (B) FLII^12^Pglu-700µδ6 nanosensors, reporting changes in intracellular PKA activity and [glucose]_i_, respectively, upon the addition of 2 mM L-lactate. Data are expressed as the percentages of FRET ratio signals relative to the baseline ratio values. The numbers in brackets are the numbers of independent experiments. Data for every set of experiment was acquired from at least two different animals.
